# P-412. Staphylococcus aureus Hand Colonization in 321 Adult Inpatients

**DOI:** 10.1093/ofid/ofae631.613

**Published:** 2025-01-29

**Authors:** Maeve Hiehle, Natasia F Jacko, Michael Z David

**Affiliations:** University of Pennsylvania, Philadelphia, Pennsylvania; University of Pennsylvania, Philadelphia, Pennsylvania; University of Pennsylvania Perelman School of Medicine, Philadelphia, Pennsylvania

## Abstract

**Background:**

*Staphylococcus aureus* (SA) hand colonization is understudied.

**Figure:**
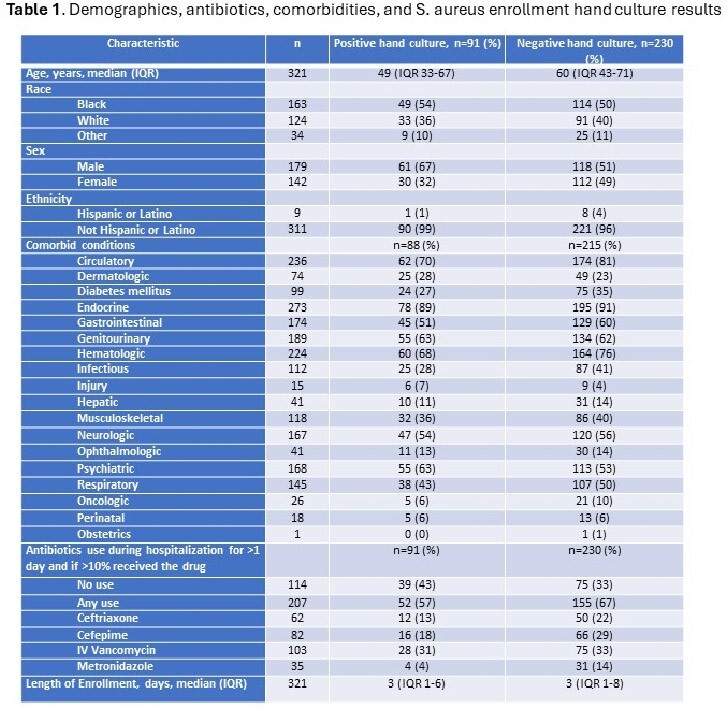

Table 1. shows the total number of subjects with each stated characteristic, as well as the number and percent of subjects with each characteristic stratified among those with positive enrollment hand cultures and those with negative hand enrollment cultures.

**Methods:**

We tested 328 adults in 2 general medicine hospital units < 3 days after admission, and for 219 of them < 2 days before discharge, for SA colonization of hands, nares, inguinal skin, and oropharynx, by direct plating and broth enrichment. Comorbidities were assessed by ICD-10 codes.

**Figure 1.**
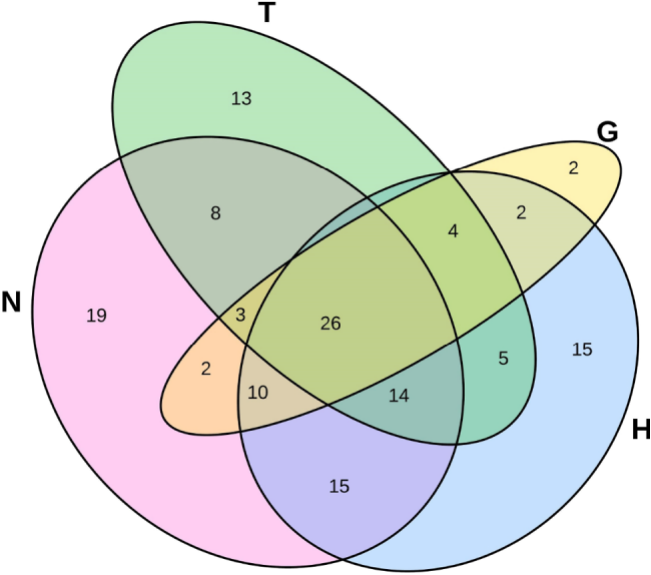
Venn Diagram showing distribution of positive S. aureus enrollment cultures from 4 body sites in 138 colonized adult inpatients

Venn diagram showing 138 of 328 (42.1%) study subjects who were found to have colonization with S. aureus at one or more of the following 4 body sites: throat (T), inguinal skin/groin (G), hand (H), or nares (N). Shows each study subject in a mutually exclusive group of single-site or multibody-site colonization. For example, 15 (4.6%) had only hand (H) colonization, while 26 (7.9%) had colonization at all 4 body sites.

**Results:**

The median age was 57.5 years (IQR 39.5-70.5), 55.8% were male, 32.9% had diabetes mellitus, 78.4% had a cardiovascular diagnosis, median study enrollment was for 3 days (IQR 1-7) and 42.1% had SA colonization at 1 or more site. 36.0% (118/328) were administered 1 or more antimicrobial drug; IV vancomycin (32%) and cefepime (25%) were most common (Table 1). SA grew from 28.4% (91/321) of enrollment and 25.6% (56/219) of discharge hand cultures.

Enrollment hand colonization (HCe) was associated with carriage at each other tested body site (p< 0.001). The nose was most (65/91, 71%) and the groin, least (42/91, 46%) commonly co-colonized with HCe (Fig. 1). Of those with groin colonization, 85% (42/49) also had HCe (Fig. 1). HCe was more common in younger patients (median 49 vs. 60 years, p=0.045) and in patients without circulatory disease (26/67, 38%, p=0.03). There was a non-significant trend toward lower rates of HCe among patients with an infectious diseases diagnosis (25/113, 22.1%, p=0.064). For those with HCe, hand colonization was present in 67% (37/55) tested at last culture, with a mean LOS of 7.6 days. Additionally, 11% (18/161) gained hand colonization at last culture (GHCLC) which was not associated with age, sex, race, any specific comorbid condition, duration of stay, nor IV vancomycin, cefepime, or metronidazole use. GHCLC was less likely for those who received ceftriaxone (0/40 vs. 17/115, p=0.007) during their hospital stay. Among 100 subjects tested at enrollment and 7 days, HCe persisted in 13/22 (59%) and was acquired by 8/78 (10%) others; 5/8 who gained hand colonization at 7 days were colonized at enrollment at another body site.

**Conclusion:**

Hand *S. aureus* colonization is common in hospitalized adults, particularly among patients who were younger, had groin colonization, and those without circulatory disease. New hand colonization occurred before discharge and was less common in those who received ceftriaxone.

**Disclosures:**

**Michael Z. David, MD PhD**, Covance: Grant/Research Support|GSK: Grant/Research Support|Johnson & Johnson: Advisor/Consultant

